# Intracellular Accumulation of Gold Nanoparticles Leads to Inhibition of Macropinocytosis to Reduce the Endoplasmic Reticulum Stress

**DOI:** 10.1038/srep40493

**Published:** 2017-02-01

**Authors:** Nuray Gunduz, Hakan Ceylan, Mustafa O. Guler, Ayse B. Tekinay

**Affiliations:** 1 Institute of Materials Science and Nanotechnology, National Nanotechnology Research Center (UNAM), Bilkent University, Ankara 06800, Turkey; 2 Max-Planck Institute for Intelligent Systems, 70569 Stuttgart, Germany; 3 Neuroscience Graduate Program, Bilkent University, Ankara 06800, Turkey

## Abstract

Understanding the toxicity of nanomaterials remains largely limited to acute cellular response, i.e., short-term *in vitro* cell-death based assays, and analyses of tissue- and organ-level accumulation and clearance patterns in animal models, which have produced very little information about how these materials (from the toxicity point of view) interact with the complex intracellular machinery. In particular, understanding the mechanism of toxicity caused by the gradual accumulation of nanomaterials due to prolonged exposure times is essential yet still continue to be a largely unexplored territory. Herein, we show intracellular accumulation and the associated toxicity of gold nanoparticles (AuNPs) for over two-months in the cultured vascular endothelial cells. We observed that steady exposure of AuNPs at low (non-lethal) dose leads to rapid intracellular accumulation without causing any detectable cell death while resulting in elevated endoplasmic reticulum (ER) stress. Above a certain intracellular AuNP threshold, inhibition of macropinocytosis mechanism ceases further nanoparticle uptake. Interestingly, the intracellular depletion of nanoparticles is irreversible. Once reaching the maximum achievable intracellular dose, a steady depletion is observed, while no cell death is observed at any stage of this overall process. This depletion is important for reducing the ER stress. To our knowledge, this is the first report suggesting active regulation of nanoparticle uptake by cells and the impact of long-term exposure to nanoparticles *in vitro*.

Advances in nanotechnology have resulted in the development of a large variety of engineered nanomaterials for numerous applications in chemistry, engineering and biomedical sciences^1^,^2^,^3^,^4^. However, the prospect of ubiquitous use of nanomaterials has also raised concerns regarding the safety of these materials^5^, so the assessment of their potential toxicities has emerged as a major research area^1^. The toxicity of nanomaterials is difficult to predict and stems from a combination of their physicochemical properties, including size^6^,^7^, morphology^7^, surface chemistry^8^, agglomeration/aggregation state^9^, solubility^10^ and biological milieu^11^, and may also vary depending on the cell types involved^12^,^13^,^14^. Therefore, both the short- and long-term effects of engineered nanomaterials should be investigated in great detail to eliminate the possibility of unforeseen health hazards prior to their use in industrial and, in particular, biomedical applications. On the other hand, the mainstream strategy for understanding the toxicity of nanomaterials remained largely limited to short-term cell viability-based assays *in vitro* and analyses of tissue-level accumulation and clearance patterns in animal models, which have produced very little information on how these materials interact adversely with profoundly complex cellular machinery^15^,^16^,^17^. Few previous reports towards this scope were mostly focused around the effects of acute toxic doses on cells occurring only in a few days, without realizing the toxicity occurring as a result of the intracellular accumulation of nanomaterials after prolonged exposure times, which is regarded vital, yet still continues to be an unexplored territory^18^,^19^.

Engineered AuNPs attract special attention because of their rather well-established fabrication methods, low variance in size and shape, convenient means of surface functionalization, high chemical stability, inertness, relative biocompatibility and low acute toxicity^20^. Consequently, AuNPs are now being used with increasing frequency in photothermal therapy^21^, targeted drug^22^ and gene delivery^23^, magnetic resonance imaging and other biomedical applications, and are also considered therapeutic agents by themselves (*e.g.* for rheumatoid arthritis)^24^. Immediately following their entry into biological systems, AuNPs interact with various local biomacromolecules to assemble a dynamic protein corona on their surfaces. The formation of this layer has significant effect on the cellular uptake^11^,^25^,^26^, targeting ability^27^, accumulation and toxicity of nanoparticles in living systems, and depends strongly on the composition of the environment encountered by the material. The long-term risk assessment of nanomaterials is typically done using animal models, which assess systemic toxicity and accumulation in organs rather than the behaviors undertaken by individual cells to reduce the local concentration of toxic material. In contrast, cell culture-based toxicity models are generally used for assessment of short-term exposure, while long-term observation is difficult because the high proliferative capacity of cells imposes constraints on maximum culture time. Thus, there is a detachment between *in vitro* and *in vivo* models of toxicity assessment and a strong need for further research into the cellular processes that are altered during long-term nanoparticle exposure. In addition, studies that do consider long-term cellular exposure tend to utilize the occupational exposure model^28^,^29^,^30^,^31^,^32^, and more studies are necessary to understand long-term nanoparticle toxicity upon continuous exposure.

In this work, we show the impact of slow, long-run intracellular accumulation of AuNPs on the nanoparticle uptake dynamics and associated intracellular stresses *in vitro*. Continuous exposure to AuNPs nanoparticles in low doses provides a reliable window for dissecting the toxicity stemming from the long-term exposure to nanoparticles, from acute toxicity resulting from high-dose exposure. The *in vitro* study model described herein is very practical and informative for the future studies regarding the accumulation of a wide scope of engineered nanomaterials.

## Results and Discussion

### Characterization of AuNPs

Citrate-capped AuNPs with *ca.* 12 nm size (12.2 ± 1.0 nm by transmission electron microscopy and 11.8 ± 2.3 nm by dynamic light scattering) were synthesized by using Turkevich method (Fig. 2B and Figure S1A). The UV-Vis spectrum of citrate-capped AuNPs displayed a distinct surface plasmon resonance peak (SPR) at 518 nm (in ultrapure water). The location of SPR signal is informative about the electronic properties of the surrounding medium and the state of nanoparticle aggregations that may potentially result from the formation of surface corona from macromolecular species. A slight red shift in the absorption spectrum of AuNPs when placed in fetal bovine serum suggested the formation of a corona on the nanoparticle surface that is caused by the displacement of weakly-bound citrate ions with multivalent macromolecules present in the serum (Fig. 2C, Figure S1B,C and D). A concomitant increase in zeta potential (from −30 mV to −10 mV) was also observed, further suggesting the alteration in the surface chemistry of the nanoparticles following introduction into the biological environment. A fluctuation in zeta potential was also observed in time-dependent measurements, which was attributable to the mobile elements of the dynamic corona (Figure S1E). Such alterations on the surface chemistry of the AuNPs suggest a new biological identity (in addition to its chemical identity determined by its size and surface characteristics) that could influence its interactions with the cells. The significance of this biological identity was further observed in the viability experiments in order to determine the exposure dose of AuNPs for accumulation studies.

### Cytotoxicity and internalization of AuNPs

Human umbilical vein endothelial cells (HUVECs) were cultured in serially-diluted concentrations of nanoparticles in 5% or 10% serum-containing media (Fig. 2D,E and F). In 5% serum containing medium AuNPs at 8 μg/mL and below concentrations were found to have no detrimental effect on the cell viability after 24 h (Fig. 2E). Higher concentrations, however, resulted in significant decrease in the cell viability because of increased nanoparticle uptake (Fig. 2E, Figures S3 and S4). In 10% serum containing medium, cell viability was *ca.* 80% at 64 μg/mL administered dose of AuNPs, whereas it was found this *ca.* 40% in 5% serum containing medium (Fig. 2D and E). Interestingly, the acute lethality of AuNPs was found to be highly dependent on the concentrations of the biomacromolecules present in the medium, especially at elevated AuNP concentrations. Therefore, interactions at the cell-nanoparticle interface appear to be strongly influenced by the altered biological identity of the AuNPs. It is well-known that the high surface energy of AuNPs allow unfolding of coronal proteins, which exposes previously unavailable domains to the biological environment^33^. The cellular uptake of nanoparticles, a very initial step towards the cytotoxic response, may then change considerably depending upon the biological environment and on the concentration of the biomacromolecular components in which the nanoparticles are presented^11^. Indeed, we observed that the amount of nanoparticles taken up by cells is considerably higher when cells were exposed to the nanoparticles in 5% serum, which is in parallel with the higher cell death caused by AuNPs in 5% serum (Fig. 2E). Consequently, the altered uptake and viability are likely to be caused by the altered characteristics of the protein corona, which is tremendously influenced by the concentration of the macromolecular species inside serum even though, both media are the same.

### *In vitro* long-term accumulation of AuNPs

**Figure 1:**
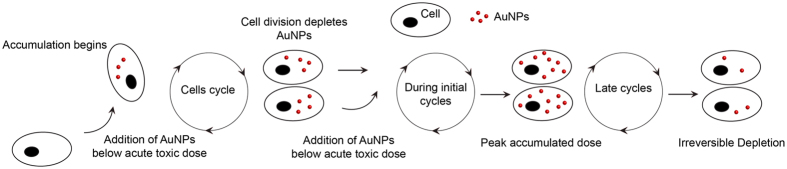
Schematic illustration of the long-term AuNP accumulation model. Endothelial cells (HUVECs) are cultivated under standard growth conditions in tissue culture plates. Additionally, their culture media were supplemented with AuNPs at a constant dose of 8 μg/mL, which is just below the acute lethal level. Based on this model, cells are expected to uptake AuNPs during the growth phase of the cell cycle, and then, typically, undergo mitosis and presumably halve their nanoparticle content into each of the offspring. Nevertheless, the continuous exposure to the nanoparticles in the medium leads to steady intracellular accumulation over time. In the early term, i.e., approximately first two weeks, cells are observed to steadily accumulate AuNPs until a maximum peak level followed by a steady decrease for the next following weeks approaching to the ground zero level.

**Figure 2:**
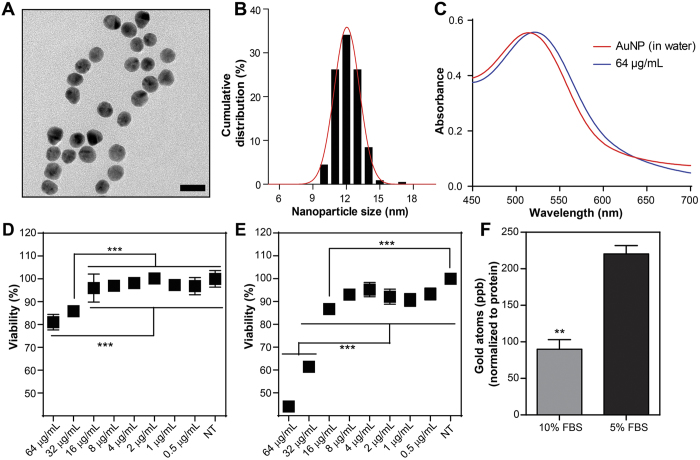
Characterizations, cytotoxicity, and protein corona effects on the internalization of AuNPs. Transmission electron micrograph (Scale bar 20 nm) (**A**) and particle size distribution of AuNPs (**B**). UV-Vis spectra of AuNPs in concentrations ranging from 64 μg/mL in 5% FBS-containing media and in water (**C**). Dose-dependent cytotoxicity in HUVECs after 24 h of AuNPs exposure in 10% FBS DMEM (**D**) and 5% FBS DMEM (**E**) (Error bars show SEM, two independent experiments were repeated with n = 4 in 10% FBS DMEM cytotoxicity and n = 8 in 5% FBS DMEM cytotoxicity experiments. One-way ANOVA with Tukey’s Multiple Comparison Test was used to show statistical significance at ***p < 0.001 and **p < 0.01). Relative uptake of 8 μg/mL AuNPs exposed in 10% FBS DMEM and 5% FBS DMEM in HUVECs after 4 h of incubation (**F**) (Error bars show SEM, two independent experiments were repeated with n = 3 in each experiment. Student’s t-test was used to show statistical significance at **p < 0.01).

**Figure 3:**
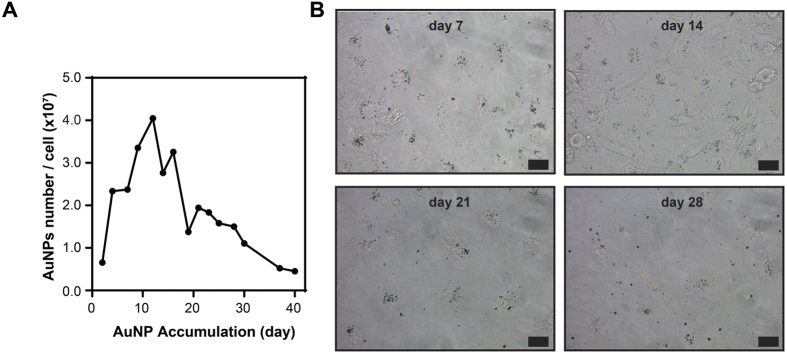
*In vitro* long-term accumulation of AuNPs in HUVECs. The amount of internalized AuNPs in HUVECs quantified from the data measured by ICP-MS at each passage and its normalization to cell number. The number of AuNPs is expressed as AuNPs per cell (**A**). Silver staining of cells at different accumulation time points (days 7, 14, 21, and 28) (**B**). Scale bars are 20 μm.

In the next step, we investigated the intracellular accumulation pattern of AuNPs in cultured HUVECs. Due to elevated uptake of AuNPs, from here on, the rest of the accumulation studies were carried out in 5% serum containing medium. HUVECs were seeded and exposed to 8 μg/mL AuNPs; after reaching ca. 90% confluency, they were subcultured and re-seeded again. This process was repeated over time to obtain long-term accumulation model (see Fig. 1 and experimental section). In order to dissect the impact of long-term nanoparticle accumulation from the acute toxic effects, the AuNPs were introduced to cells below the acute lethal dose, which was determined as 8 μg/mL from the viability assay (Fig. 2E,F). During the first two weeks, AuNPs rapidly accumulated inside HUVECs (Fig. 3A). Intracellular accumulation was further evidenced by silver staining, which enables visualization of the AuNPs under light microscope. Independent repeats of this experiment showed that this initial accumulation period could extend until 20 days, with the same pattern in each repeat. Nonetheless, no further accumulation was observed after this period. Strikingly, the peak accumulation is followed by a steady decline in the amount of intracellular AuNPs (Fig. 3B). Furthermore, we observed repeatable fluctuations in the intracellular AuNPs amount even though the overall trend is down, during the decline period (Fig. 3A). There are two possible explanations for the decline in the intracellular AuNP accumulation: (a) the uptake of new AuNPs is inhibited by inhibiting the endocytosis process, or (b) AuNPs inside the cells are actively transported from the cells to the outside environment. There are few reports on the exocytosis of surface-functionalized AuNPs^34^,^35^,^36^ despite the fact that inhibition of the uptake process has not been previously reported.

### Endocytosis and exocytosis behavior of AuNP accumulated cells

To understand endocytosis and exocytosis behavior, we first investigated the mechanism of AuNPs uptake by HUVECs. Although the uptake mechanisms of bare and surface-functionalized AuNPs in a variety of sizes and shapes have been characterized in the literature, their internalization is difficult to generalize and may depend on processes such as macropinocytosis^37^, phagocytosis^38^,^39^, receptor mediated endocytosis^7^ (clathrin- or caveolae-mediated endocytosis) and transcytosis^40^. Therefore, three of these major pathways (macropinocytosis, caveolae-mediated endocytosis, and clathrin-mediated endocytosis) were blocked by inhibitor treatment to observe whether AuNP uptake depended on any of these mechanisms (Table S1). The uptake of AuNPs was observed to greatly (*ca.* 30%) diminish following the inhibition of macropinocytosis while this decrease was not as pronounced after inhibition of caveolae- or clathrin-mediated endocytosis (Figure S2). This result suggests that AuNPs were predominantly entering into HUVECs via macropinocytosis. Gulsuner et al, showed that MDP-AuNPs which contains RGD sequence as targeting moiety enters into breast cancer cells via both clathrin-mediated endocytosis and macropinocytosis pathway^37^. The cells might use non-specific entry routes for endocytosis of non-functionalized AuNPs. Therefore, lack of targeting moiety might activate macropinocytosis pathway for nanoparticle endocytosis.

In order to understand if any of these major routes of uptake is impaired during the accumulation process, we investigated the endocytosis competency of cells on day 23, one of the early days of the decline in the intracellular AuNP amount (Fig. 3A). For this purpose, cells were incubated with fluorophore-conjugated molecules that enter cells through specific endocytosis pathways and then checked if these molecules are inside the cells. Among these molecules, choleratoxin enters cells through caveola-mediated endocytosis, transferrin enters via clathrin-mediated endocytosis, and dextran enters via macropinocytosis. While cholera toxin and transferrin entries were observed in both AuNP (+) and AuNP (−) groups, dextran entry was observed only in the AuNP (−) group, indicating that macropinocytosis, the main route of AuNP uptake, ceased altogether (Fig. 5). Nevertheless, the complete inhibition of macropinocytosis is far from explaining the drastic decrease in the intracellular AuNP. As a result, we further explored a potential role for exocytosis for the active transport of AuNPs out of the cells, however, we detected no evidence that exocytosis plays a significant role for the removal of AuNPs inside the cells (Figure S5). It is likely that cell division plays a predominant role for dilution of AuNPs in the new offspring cells^41^. Because the macropinocytosis is inhibited, these two effects are combined to culminate in a gradual decrease of AuNPs inside the cells.

### Determination of cellular stress during long-term accumulation

**Figure 4:**
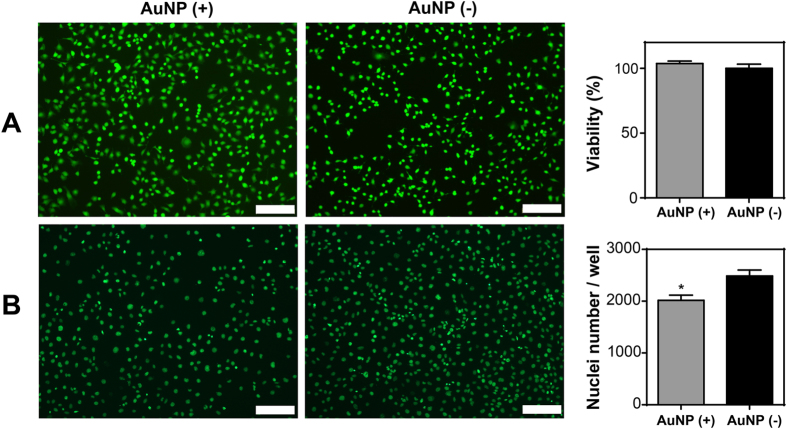
Cellular response to long-term accumulation of AuNPs. Cytotoxicity of AuNPs after 57 days was assessed with Live/Dead (Calcein Am/Ethidium homodimer staining) and MTT assay. Live cells were stained green and dead cells were stained red. Student t-test was used for statistical analysis and significance was not found (**A**). Proliferation of cells after 49 days of accumulation was assessed with Click-iT EdU assay. The nuclei of proliferating cells were stained green. Student t-test shows statistical significance with *p < 0.05 (**B**). Error bars show SEM in all graphics, experiments were repeated with n = 3 in both viability and proliferation experiments. Scale bars are 200 μm in all images.

**Figure 5:**
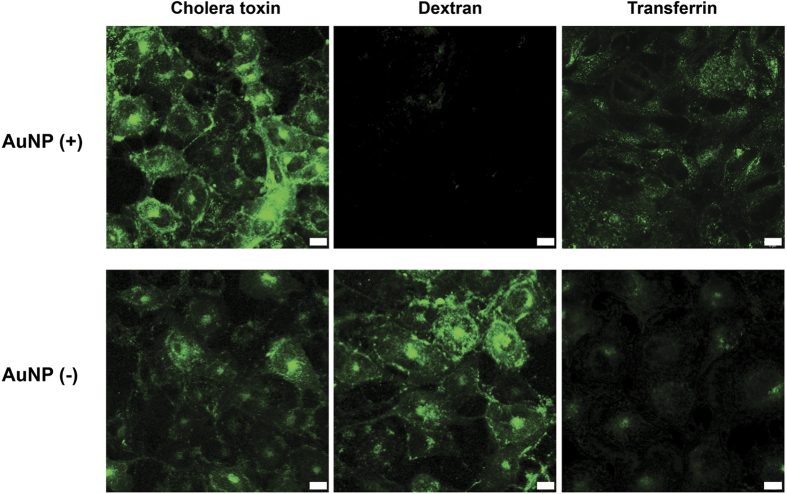
Uptake profile of HUVECs (at day 23) incubated with fluorescence-labeled compounds that enter cells through specific endocytosis pathways. Cholera toxin enters through caveolin-mediated endocytosis, dextran enters through macropinocytosis, and Transferrin enters through clathrin-mediated endocytosis. Scale bars are 10 μm.

**Figure 6:**
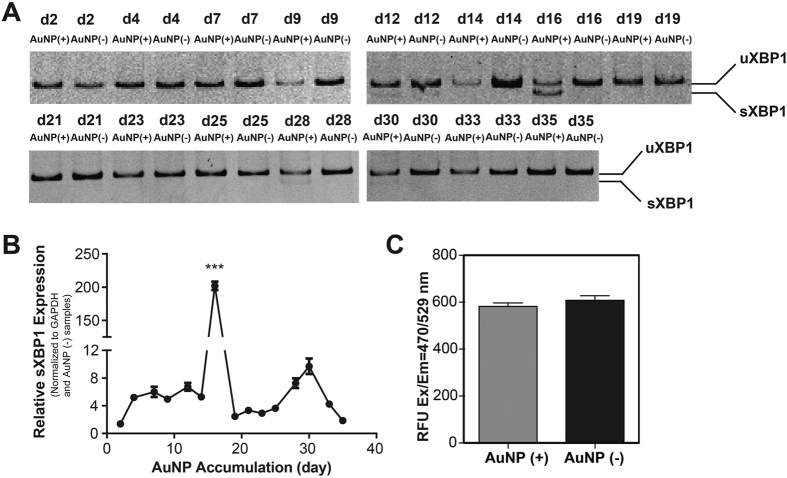
Cellular stress response to long-term accumulation of AuNPs. RT-PCR analysis of an ER stress indicator (XBP1) in HUVECs at different AuNP accumulation time points following 10% polyacrylamide gel electrophoresis. The unspliced uXBP1 (289 bp) and spliced sXBP1 (263 bp) forms of XBP1 are indicated (**A**). qRT-PCR analysis of spliced XBP1 (sXBP1) in HUVECs at different accumulation time points, where expression of sXBP1 was normalized to GAPDH and AuNP (−) samples. One way ANOVA with Dunnett’s Multiple Comparison test was used to show statistical significance at ***p < 0.001 (**B**). ROS levels were analyzed in AuNP (+) and AuNP (−) cells after 57 days of accumulation by DCF assay (**C**) (Error bars show SEM, experiment was performed with n = 3. Student t test was applied for statistical analysis).

Engineered nanomaterials bound to biomacromolecules can cause conformational changes^42^ in the structure of proteins, which might result in ER stress through the unfolded protein response (UPR), and trigger oxidative stress^10^ through ROS formation. ER stress is generally considered an adaptive reaction of cells to external stress, and serves as a survival mechanism. This complex stress response is mediated by several molecules, such as PKR-like ER kinase (PERK), activated transcription factor 6 (ATF6), inositol-requiring enzyme 1 (IRE1), and spliced X box-binding protein 1 (sXBP1)^43^. IRE1 is activated in response to accumulation of unfolded proteins, and acts as a site-specific endonuclease splicing out a 26-nucleotide intron in XBP1 mRNA. Spliced XBP1 mRNA is translated to produce spliced XBP1 protein, which is a transcription factor and one of the key regulators of the protein-folding capacity of the cells and ER homeostasis^43^. Therefore, cellular levels of XBP1 and its spliced form, sXBP1 were monitored through polyacrylamide gel electrophoresis (PAGE) and qRT-PCR to evaluate ER stress at different accumulation time points (Table S2). Interestingly, a slight sXBP1 band was observed at the point of highest accumulation (day 12), which became more pronounced at day 16 in the AuNP (+) group but not observed in the AuNP (−) group (Fig. 6A). Moreover, there are also slight bands at day 28 and 30 in the AuNP (+) group but not seen in the AuNP (−) group. This result was also confirmed using only the sXBP1 primer in qRT-PCR, where a sharp peak with significant increase of 201.94 ± 6.29 of AuNP (−) group was seen at day 16. sXBP1 expression was statistically significant to other AuNP accumulated days (Fig. 6B). Expression profile of sXBP1 correlates with accumulation of AuNPs inside the cells, which suggests that peak accumulation occurs at day 12 due to slight ER stress, while increased ER stress at day 16 triggers a decline in AuNP accumulation. Under prolonged ER stress, cells typically ignite a specific cell death program, apoptosis^44^. At the end of 57 days of accumulation, both viability and ROS levels in AuNP (+) and AuNP (−) cells were comparable, indicating that long-term accumulation of AuNPs did not induce cell death and ROS formation (Figs 4A and 5C). Thus, it is likely that cells exposed to AuNPs for longer periods develop a survival mechanism via short-term on/off of ER stress followed by inhibition of their macropinocytosis pathway, and thereby control the intracellular concentration of AuNPs. A link was previously established between ER stress and endocytosis inhibition in the literature^45^, and this cooperative mechanism might possibly serve to control intracellular concentration of engineered nanomaterials. On the other hand, the proliferative capacity of cells in AuNP (+) group decreased slightly but significantly when compared to AuNP (−) cells (Fig. 4B). One week exposure of low dose of citrate-capped AuNPs (~18 nm in diameter) has previously been reported to trigger a similar decrease in cell proliferation without compromising cell viability^46^.

## Conclusion

In this work, we show the toxic impact of chronic exposure and accumulation of AuNPs on endothelial cells. Even though AuNPs are largely considered “non-toxic” and “safe” based on the short-term exposure assays, we realize that their long-term interaction with the cellular machinery is highly complex and creates major challenges for cells to eliminate the stress caused by its accumulation. To elucidate this, we designed a new *in vitro* accumulation model, which enables assessment at non-lethal exposure doses over an extended period of time. This platform emulates certain aspects of *in vivo* exposure conditions as well as utilizes a simple-to-implement cell culture model that can be used to directly analyze the long-term effects of other types of nanoparticles accumulated in the cellular environment. The information deduced from the accumulation experiments demonstrates the strength of the model, thereby paving the way of improving risk assessment methods and extending protocols for the development of alternate cell-based models to better understand the nanomaterial safety and the associated risks. Nevertheless, there are still important challenges and questions that remain to be addressed for the optimal evaluation of long-term nanomaterial toxicity. For example, a more robust method could be developed where cell division and proliferation is inhibited, so the effect of cell division on diluting the intracellular nanoparticles is eliminated. However, the challenge here is to keep cells alive and healthy under the cell cycle arrest conditions for extended periods of time. Ultimately, this and other complementing *in vitro* toxicity models are expected to provide invaluable mechanistic information about cell-nanomaterial interactions at the molecular level as well as enormous practical flexibility for experimental setup design, thereby significantly reducing the need for animals-based models and number of animals being sacrificed.

## Experimental Section

Chemicals and Reagents. Gold (III) chloride trihydrate (HAuCl_4_.H_2_O), 2′, 7′ dichlorofluorescein diacetate, 3-(4, 5-dimethylazol-2-yl)-2, 5-diphenyl-tetrazolium bromide (MTT) kit, Nystatin, Amiloride hydrochloride hydrate, Chlorpromazine hydrochloride, Silver Enhancer Kit SE-100, hydrocholoric acid fuming 37%, and Nitric acid >65% were purchased from Sigma-Aldrich Chemical Co. Ethidium homodimer, Calcein Am, Click-iT EdU assay, TRIzol, Alexa Fluor 488 conjugated Cholera toxin subunit B (recombinant), Fluorescein conjugated Dextran (10000 MW) and Alexa Fluor 488 conjugated Transferrin from human serum were purchased from Invitrogen (C34775, D1820, and T13342). All cell culture materials were also purchased from Gibco, Invitrogen. Sodium citrate tribasic were obtained from Codex, Carlo Erba. Amicon^®^ Ultracel centrifugal falcons were purchased from Millipore. Ultrapure water (18.2 MΩ) was obtained from a Sartorios arium^®^ Lab Water System.

### Synthesis of AuNPs

All glassware was carefully cleaned with aqua regia (3:1 mixture of hydrochloric and concentrated nitric acids), and rinsed with distilled water several times before use. AuNPs with 12-nm diameter were synthesized according to the previously published Turkevich protocol^47^. Briefly, 100 mL of HAuCl_4_ (1 mM) in water was boiled under stirring, and 10 mL of sodium citrate trihydrate solution (38.8 mM) was added rapidly to the chloride solution, which resulted in a change in solution color from pale yellow to deep red.

### UV-Vis Spectroscopy and Transmission Electron Microscopy

UV-Vis spectra of AuNP solutions were measured using a SpectraMax M5 Multi-Mode Microplate Reader. Spectra of freshly prepared AuNPs and water (for baseline subtraction) were collected using quartz cuvettes with 1 cm optical path lengths and in a 250–750 nm range with 2 nm increments. Structural characterization of the AuNPs was performed using a FEI Tecnai G2 F30 transmission electron microscope (TEM) at 300 kV. TEM samples were prepared on a fine mesh Cu grid by incubating a drop of AuNP solution for 5 min, washing with a drop of distilled water for 2 min and repeating these steps for two more times prior to drying. Individual particle sizes were measured using ImageJ (NIH) software. After the characterization of the nanoparticles by TEM and UV-Vis spectroscopy, their initial concentration was calculated by using previously established theoretical calculations^48^. The nanoparticles were then concentrated by using Amicon^®^ Ultracel centrifugal falcons (10 K) at 6000 rpm for 5 min (repeated multiple times for required concentrations).

### Dynamic Light Scattering (DLS) and Zeta Potential Measurements

DLS measurements were carried out using a Malvern NanoZS Zetasizer at room temperature. Auto-correction was done as the average of three runs of 10 s each (Malvern Instruments, Southborough, MA).

### Cell Culture and Maintenance

Human umbilical cord vein endothelial cells (HUVECs) were donated by Yeditepe University, Istanbul, Turkey. HUVECs were purified as described^49^ and were characterized by staining with CD34, CD31, and CD90 surface markers. These cells were found to be positive for CD31 and CD34 but negative for CD90. HUVECs were cultured in a humidified, 37 °C, 5% CO_2_ incubator using 75 cm^2^ polystyrene cell culture tissue flasks containing Low Glucose Dulbecco’s modified Eagle’s medium (DMEM) supplemented with 5% or 10% heat inactivated fetal bovine serum (FBS, Gibco), 1% penicillin/streptomycin (P/S) and 2 mM L-glutamine. Passaging of cells was performed at cell confluency between 90 to 100% using trypsin/EDTA. Cells were diluted 1:4 for subculturing.

### Cell Viability and Proliferation

HUVECs were seeded at a density of 7.5 × 10^3^ cells/well in a 96-well plate. After 4 h of incubation, media were removed and replaced with either 5% FBS or 10% FBS DMEM containing AuNPs at concentrations ranging from 0.5 μg/mL to 64 μg/mL, and cells were incubated for 24 h at 37 °C in a humidified incubator with 5% CO_2_. At the end of the exposure period, the toxicity of AuNPs was assessed by a standard colorimetric cellular viability assay, in which 3-(4,5-dimethylazol-2-yl)-2,5-diphenyl-tetrazolium bromide (MTT) dye is converted to insoluble purple formazan crystals and metabolic activity is evaluated through absorbance measurements at 570 nm using a SpectraMax microplate reader (Molecular Devices, Sunnyvale, CA). All experiments were performed in quadruplicate and with three independent repeats.

Proliferative cells were determined using Click-iT EdU assay (Molecular Probes). HUVECs were incubated with a nucleoside analogue of thymine, EdU (5-ethynyl-20-deoxyuridine), in the culture medium. EdU is incorporated into DNA during the synthesis phase (S phase) of the cell cycle, and hence enables direct quantification of proliferation. HUVECs were seeded on 15 mm cover glasses at a density of 2.5 × 10^4^ cells/glass. After 8 h of incubation, one half of the medium was replaced with 20 μM EdU-containing fresh media supplemented with 5% FBS. Cells were then post-incubated for 48 h for the incorporation of the thymine analogue to DNA. Following post-incubation, cells were fixed with 4% paraformaldehyde, permeabilized with 0.5% Triton-X, and treated with Alexaflour-488 conjugated azide as recommended by the supplier. Proliferative cells were imaged by fluorescent microscopy. Stained cell nuclei were counted with ImageJ (NIH) software. The average counts of stained cell nuclei were used to evaluate the relative proliferative cell numbers. Both viability and proliferation results were normalized to non-treated control group.

### *In Vitro* Long-term Accumulation Model

Following the assessment of cell viability, a non-toxic dose of 8 μg/mL was chosen for long-term accumulation experiments. HUVECs were seeded in 6 well plates at a density of 1 × 10^5^ cells/well, and exposed to AuNPs after their attachment to well surfaces (~4 h). HUVECs were later subcultured after reaching *ca.* 90% confluency and re-seeded again at 1 × 10^5^ cells/well density to 6-well plates for further incubation. This process was repeated in three day intervals, as represented in Fig. 1. In each passage, the remaining cell pellets were used for RNA isolation, protein isolation, viability, and proliferation analyses, silver staining and ICP-MS measurements. For ICP-MS measurements, cells were counted at least three times with trypan blue to determine the remaining cell amounts for normalization.

### Imaging of AuNPs within Cells by Silver Staining

HUVECs were seeded on 13 mm cover glasses at a density of 2.5 × 10^4^ cells/glass. After 4 h of incubation, media were removed and replaced with media containing AuNPs at concentrations ranging from 0.5 μg/mL to 64 μg/mL, and the cells were incubated for 24 h. Cells were then washed three times with 1x PBS to remove excess AuNPs. After washing, cells were fixed with 4% paraformaldehyde for 15 min, and silver staining was performed using Sigma Silver Enhancer Kit according to the manufacturer’s instructions. Bright field images were taken after staining to visualize AuNP presence within cells. Silver staining in different long-term accumulation time point was also performed with identical protocol explained above.

### Quantification of AuNPs within Cells by ICP-MS

To investigate protein corona effects on the internalization of AuNPs, short-term uptake of AuNPs in 5% FBS DMEM and 10% FBS DMEM was performed in HUVECs. The cells were seeded in 6 well plates at a density of 1 × 105 cells/well, and exposed to 8 μg/mL AuNPs in 5% FBS DMEM and 10% FBS DMEM after their attachment to well surfaces (~4 h). After 4 h incubation with AuNPs, cells were washed three times with 1x PBS to remove excess AuNPs. After washing, cells were detached from the surfaces with trypsinization and collected with centrifugation at 2500 rpm for 5 min. The protein isolation was performed according to instructions of M-PER^®^ Mammalian Protein Extraction Kit (Thermo Scientific). Protein concentrations of samples were determined with Pierce™ BCA Protein Assay (Thermo Scientific). Then, samples were digested by aquaregia, and diluted in 2% nitric acid immediately prior to measurement. A five-point calibration curve was prepared in a gold concentration range of 1.25–20 ng/mL, and the quantification was performed through linear regression of the measured intensity of the analyte and internal standard (Bi) versus the concentration of calibration standards. All results were reported in ppb or ppm of gold per sample. Gold measurements were performed using a Thermo Scientific X Series 2 ICP-MS.

To investigate long-term accumulation of AuNPs in HUVECs over time, cells were counted at least three times with trypan blue after each passage to determine the remaining cell amounts for normalization. After that, all cell pellets were kept frozen until analysis, and before analysis they were thawed. The following procedure for ICP-MS measurement was performed same as mentioned above. The number of AuNPs per cell was calculated as:


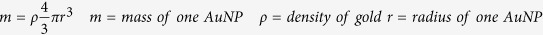


To obtain number of AuNP in each sample, total ppb determined by ICP-MS was divided to the mass of one AuNP. Total AuNP number was divided to cell number.

### Imaging of HUVECs with Fluorescent Molecules for Endocytosis Analysis

At day 23 of long-term accumulation, HUVECs were seeded on 13 mm cover glasses at a density of 2.5 × 10^4^ cells/glass. After 4 h of incubation, media were removed and replaced with media containing 5 μg/mL Alexa Fluor 488 conjugated Cholera toxin subunit B (recombinant), 100 μg/mL Fluorescein conjugated Dextran (10000 MW) and 25 μg/mL Alexa Fluor 488 conjugated Transferrin from human serum and the cells were incubated additional 4 h. Cells were then washed three times with 1x PBS to remove excess molecules. After washing, coverslips were closed on glass slides and immediately imaged with Zeiss LSM 510 Meta before cell death.

### Quantification of AuNPs in Medium by ICP-MS for Exocytosis Analysis

During long-term accumulation experiment, all media were collected for exocytosis analysis before AuNPs exposure to the cells. Briefly, HUVECs were seeded in 6 well plates at a density of 1 × 10^5^ cells/well, and medium was collected for ICP-MS measurement and replaced with AuNPs containing medium after their attachment to well surfaces (~4 h). All collected medium samples were kept frozen until analysis, and the following procedure for ICP-MS measurement was performed same as mentioned above.

### Determination of Cellular Internalization Pathway of AuNPs

Endocytosis inhibitors are often used to determine which endocytosis pathway plays a central role for cellular uptake of NPs^50^. The concentrations and treatment times of small molecule chemical inhibitors were optimized in a preliminary experiment to select the maximum non-toxic doses and treatment times. HUVECs were seeded at 2.5 × 10^4^ cells/well density in 24 well plates. After 4 h of incubation, cells were treated with chemical inhibitors at final concentrations of 2.5 mM amiloride, 100 μg/mL nystatin and 2.5 μg/mL chlorpromazine to inhibit specific pathways and to understand the internalization mechanism of AuNPs (Table S1). After 1 h incubation, the inhibitors were removed, and the cells were exposed to 8 μg/mL AuNPs in 5% FBS DMEM. After 4 h incubation with AuNPs, cells washed three times with 1x PBS to remove excess AuNPs. After washing, cells were detached from the surfaces with trypsinization and collected with centrifugation at 2500 rpm for 5 min. The protein isolation was performed according to instructions of M-PER^®^ Mammalian Protein Extraction Kit (Thermo Scientific). After that protein concentrations of samples were determined with Pierce™ BCA Protein Assay (Thermo Scientific). Then, ICP-MS measurement was performed same as mentioned above.

### Determination of Cellular Stress Levels by qRT-PCR and Dichlorofluorescein assay

RT-PCR and qRT-PCR were used to quantify XBP1 and sXBP1 amounts. Total RNA was isolated from remaining cell pellets using TRIzol (Invitrogen) according to manufacturer’s instructions. Nanodrop 2000 (Thermo Scientific) was used to assess the yield and purity of extracted RNA. Primer sequences are listed in Table S2. Reaction efficiencies for each primer set were evaluated with standard curves using 2-fold serial dilutions of total RNA. cDNA synthesis from RNA and RT-PCR were performed using SuperScript III One-Step RT-PCR Kit (Invitrogen) according to manufacturer’s instructions. PCR products of XBP1 were electrophoresed on 10% PAGE at 80 V for 5 h. The size difference between spliced and unspliced XBP1 is 26 nucleotides. sXBP1 gene expression profiles were analyzed at each time point of accumulation. Reaction conditions were briefly as follows: 55 °C for 5 min, 95 °C for 5 min, 40 cycles of 95 °C for 15 s, 60 °C for 30 s, and 40 °C for 1 min, followed by a melting curve to confirm product specificity. For gene expression analysis a comparative Ct method was used with formula of (primer efficiency)^−ΔΔCt^ where ΔΔCt and ΔCt were explained as:


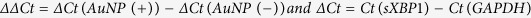


Reactive oxygen species (ROS) formation was analyzed with Dichlorofluorescein (DCF) assay after 57 days of accumulation.

### Statistical Analysis

All experiments were independently repeated at least twice, with at least four replicas for each experimental or control group in each independent assay. All quantitative results were expressed as means ± SEM. Statistical analyses were carried out by one-way analysis of variance (ANOVA) or Student’s *t* test, whichever applicable. Degrees of significance are indicated in graphs when necessary (*p < 0.05; **p < 0.01; ***p < 0.001).

## Additional Information

**How to cite this article**: Gunduz, N. *et al*. Intracellular Accumulation of Gold Nanoparticles Leads to Inhibition of Macropinocytosis to Reduce the Endoplasmic Reticulum Stress. *Sci. Rep.* **7**, 40493; doi: 10.1038/srep40493 (2017).

**Publisher's note:** Springer Nature remains neutral with regard to jurisdictional claims in published maps and institutional affiliations.

## Supplementary Material

Supplementary Information
