# Supplementary material for: Intracellular Accumulation of Gold Nanoparticles Leads to Inhibition of Macropinocytosis to Reduce the Endoplasmic Reticulum Stress
Source: Sci Rep. 2017 Feb 1;7:40493. doi: 10.1038/srep40493 (PMC5286442; doi:10.1038/srep40493)
Supplement: Supplementary Information [file srep40493-s1.doc]

**Intracellular Accumulation of Gold Nanoparticles Leads to Inhibition of Macropinocytosis to Reduce the Endoplasmic Reticulum Stress**

**Authors:** Nuray Gunduz^⁋†^, Hakan Ceylan^†‡^, Mustafa O. Guler^⁋^* and Ayse B. Tekinay^⁋§^*

**Author Affiliations**

^⁋^Institute of Materials Science and Nanotechnology, National Nanotechnology Research Center (UNAM), Bilkent University, Ankara, Turkey 06800

^‡^Max-Planck Institute for Intelligent Systems, 70569 Stuttgart, Germany

^§^Neuroscience Graduate Program, Bilkent University, Ankara, Turkey 06800

*Corresponding Author E-mail Address:

moguler@unam.bilkent.edu.tr (M.O.G.) and atekinay@bilkent.edu.tr (A.B.T.)

Tel: +90 312 290 8985, Fax: +90 312 266 4365

^†^ Equal contribution to the present manuscript

**
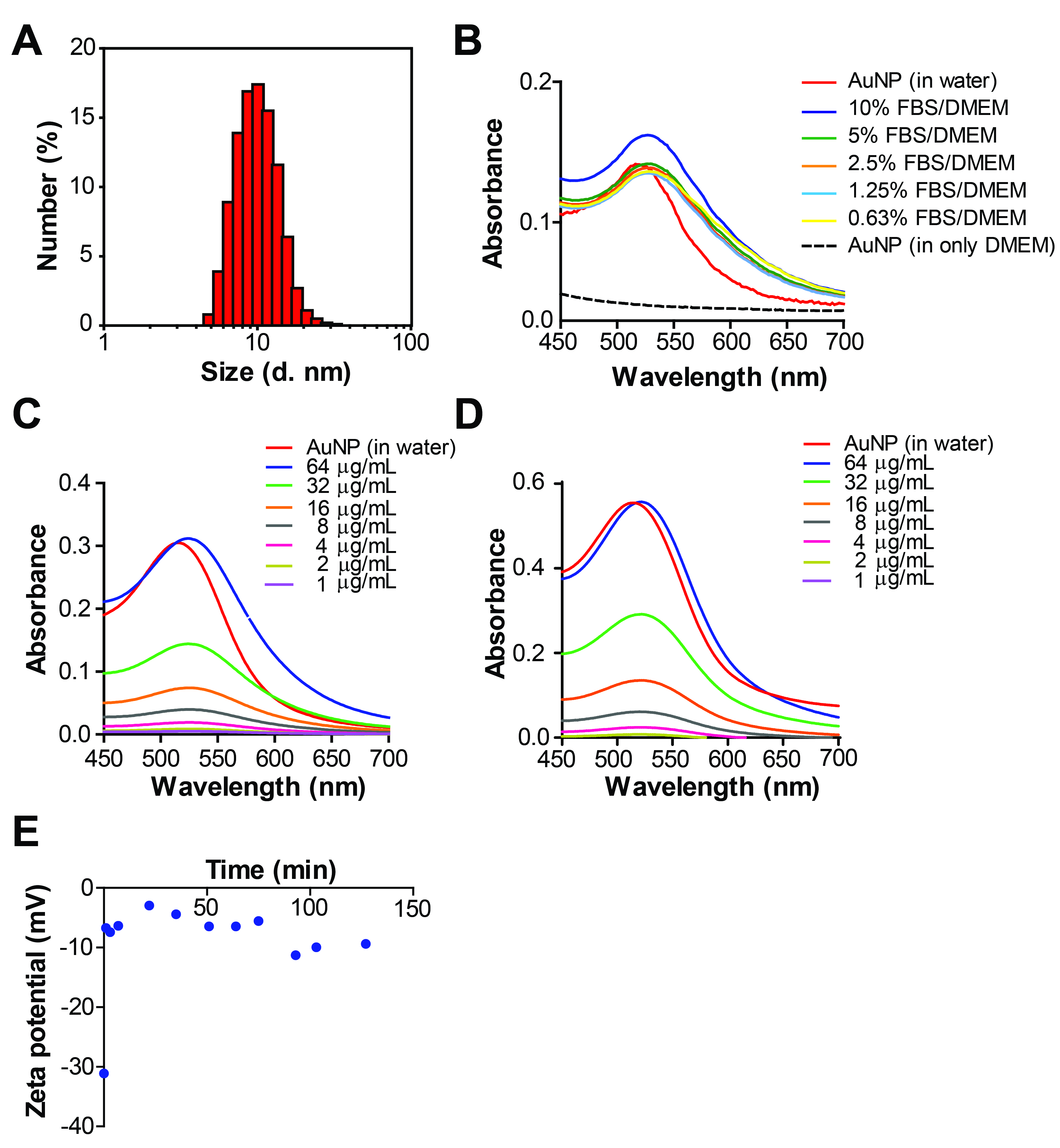
**

**Figure S1.** Characterization of AuNPs. Particle size distribution in dynamic light scattering (DLS) (A), colloidal stability of AuNPs in media containing different amounts of FBS (B), UV-vis spectra of AuNPs in a concentration range of 1-64 µg/mL in 10% FBS containing media (C), UV-vis spectra of AuNPs in a concentration range of 1-64 µg/mL in 5% FBS containing media (D), and zeta potential of AuNPs in 5% FBS containing media (E).


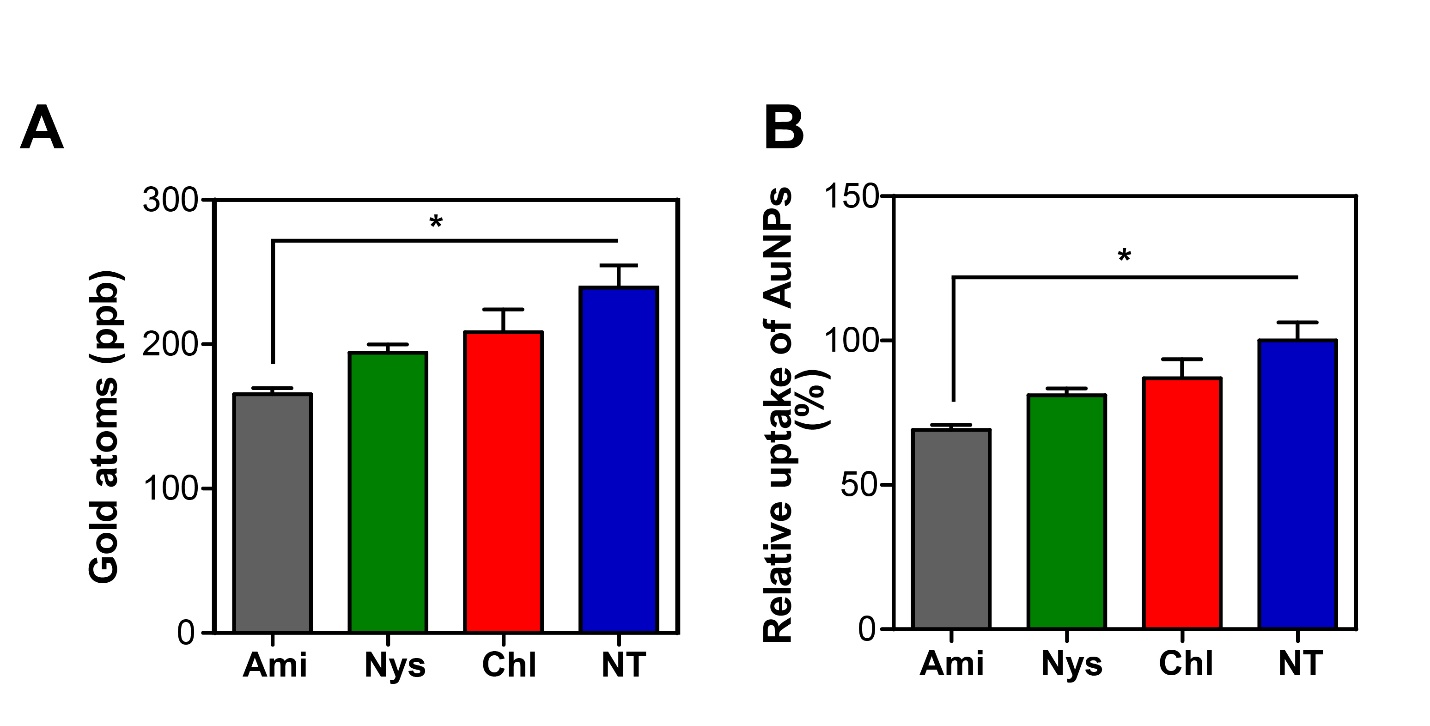


**Figure S2.** Cellular uptake of AuNPs. ICP-MS analysis of HUVECs treated with chemical inhibitors of endocytosis and incubated with 8 μg/mL of AuNPs. After 4 h of incubation, total gold atoms (A) and relative uptake (B) in HUVECs were calculated by normalization to protein amounts and untreated (NT) group. (Error bars show SEM, two independent experiments were repeated with n=3 in each experiment. One-way ANOVA with Tukey’s Multiple Comparison Test was used to show statistical significance at *p < 0.05.) (Amiloride blocks macropinocytosis; chlorpromazine prevents the formation of clathrin pits and nystatin disrupts caveolae function).


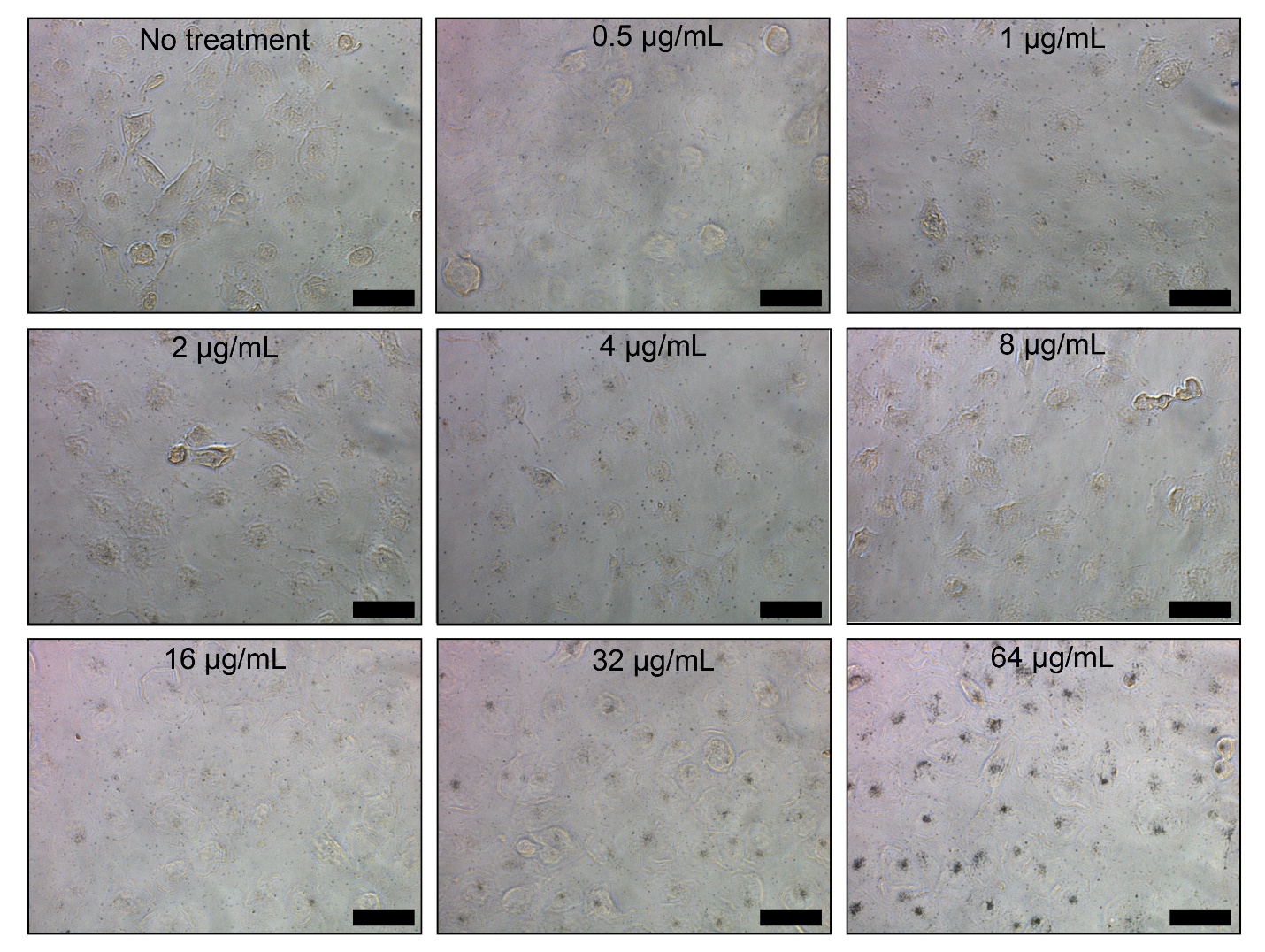


**Figure S3.** Dose dependent silver staining of HUVECs after 24 h of incubation with increasing concentrations of AuNPs in 10% FBS DMEM. Scale bars are 50 µm.


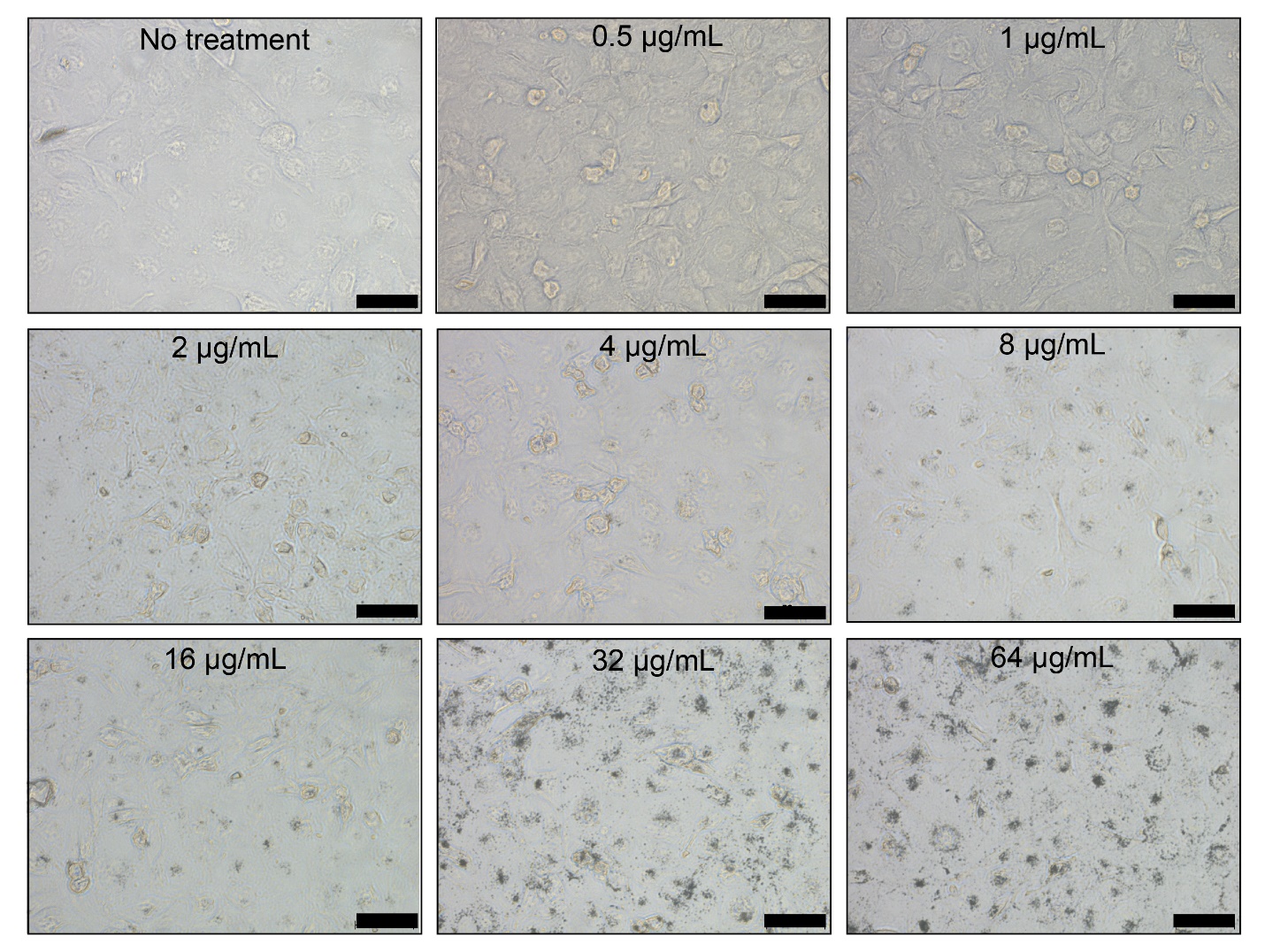


**Figure S4.** Dose dependent silver staining of HUVECs after 24 h of incubation with increasing concentrations of AuNPs in 5% FBS DMEM. Scale bars are 50 µm.


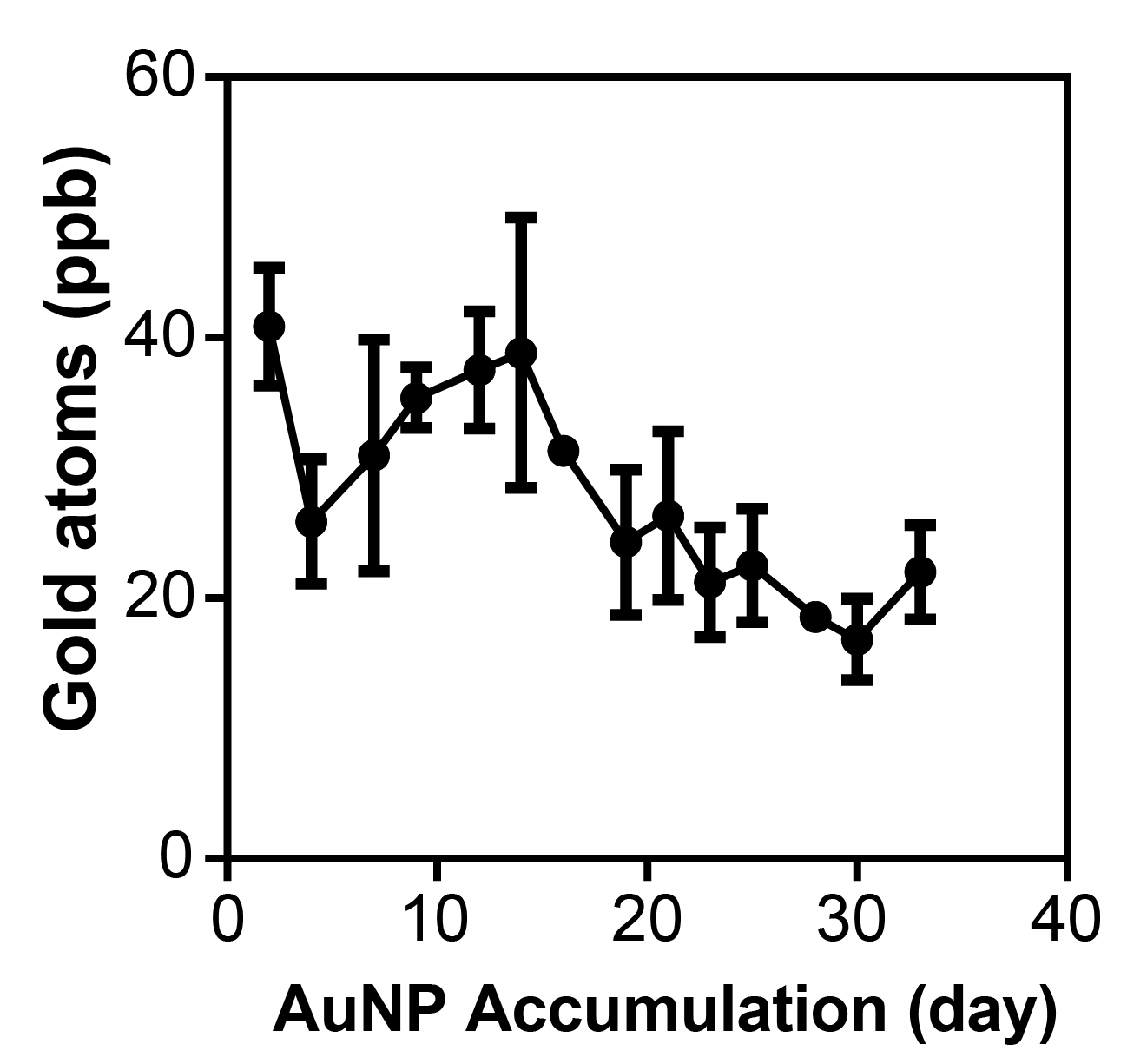


**Figure S5.** Exocytosis behavior of HUVECs during long-term accumulation of AuNPs. ICP-MS analysis of total gold atoms in medium collected before AuNP exposure. (Error bars show SEM, experiments were performed with n=3.)

Table S1. Chemical inhibitors used for inhibition of endocytosis pathway of cells.

| **Inhibitor** | **Amiloride** | **Nystatin** | **Chlorpromazine** |
| --- | --- | --- | --- |
| Clathrin | No | No | Yes |
| Caveolin | No | Yes | No |
| Macropinocytosis | Yes | No | No |

Table S2. Primers used in RT-PCR and qRT-PCR.

| Gene | Forward Primer | Reverse Primer |
| --- | --- | --- |
| XBP1 ^1^ | 5’TTACGAGAGAAAACTCATGGCC3’ | 5’GGGTCCAAGTTGTCCAGAATGC3’ |
| sXBP1 ^2^ | 5’TGCTGAGTCCGCAGCAGGTG3’ | 5’GCTGGCAGGCTCTGGGGAAG3’ |
| GAPDH | 5’GTCAAGCTCATTTCCTGGTATG3’ | 5’CTCTCTTGCTCAGTGTCCTTG3’ |

**References**

1 Samali, A., Fitzgerald, U., Deegan, S. & Gupta, S. Methods for monitoring endoplasmic reticulum stress and the unfolded protein response. *Int J Cell Biol* **2010**, 830307, doi:10.1155/2010/830307 (2010).

2 van Schadewijk, A., van't Wout, E. F., Stolk, J. & Hiemstra, P. S. A quantitative method for detection of spliced X-box binding protein-1 (XBP1) mRNA as a measure of endoplasmic reticulum (ER) stress. *Cell stress & chaperones* **17**, 275-279, doi:10.1007/s12192-011-0306-2 (2012).
